# Associations of *IL-4*, *IL-4R*, and *IL-13* Gene Polymorphisms in Coal Workers' Pneumoconiosis in China: A Case-Control Study

**DOI:** 10.1371/journal.pone.0022624

**Published:** 2011-08-03

**Authors:** Meilin Wang, Shasha Wang, Zhifang Song, Xiaomin Ji, Zhengdong Zhang, Jianwei Zhou, Chunhui Ni

**Affiliations:** 1 Department of Occupational Medicine and Environmental Health, School of Public Health, Nanjing Medical University, Nanjing, China; 2 General Hospital of Xuzhou Mining Business Group Co Ltd., Xuzhou, China; Finnish Institute of Occupational Health, Finland

## Abstract

**Background:**

The *IL-4*, *IL-4* receptor (*IL4R*), and *IL-13* genes are crucial immune factors and may influence the course of various diseases. In the present study, we investigated the association between the potential functional polymorphisms in *IL-4*, *IL-4R*, and *IL-13* and coal workers' pneumoconiosis (CWP) risk in a Chinese population.

**Methods:**

Six polymorphisms (C-590T in *IL-4*, Ile50Val, Ser478Pro, and Gln551Arg in *IL-4R*, C-1055T and Arg130Gln in *IL-13*) were genotyped and analyzed in a case-control study of 556 CWP and 541 control subjects.

**Results:**

Our results revealed that the *IL-4* CT/CC genotypes were associated with a significantly decreased risk of CWP (odds ratio (OR) = 0.74, 95% confidence interval (CI) = 0.58–0.95), compared with the TT genotype, particularly among subgroups of age <65 years (OR = 0.68, 95%CI = 0.46–0.99) and dust exposure years ≥26 years (OR = 0.69, 95%CI = 0.50–0.94). Moreover, the polymorphism was significantly associated with risk of CWP patients with stage I. In addition, a combined effect was observed in a dose-dependent manner with increasing numbers of risk variant alleles (*P*_trend_ = 0.023), and individuals with 11–12 risk alleles had a 47% higher risk of CWP than those with 0–8 risk alleles (OR = 1.47, 95% CI = 1.05–2.05).

**Conclusions:**

Our results suggest that the *IL-4* C-590T polymorphism is involved in the etiology of CWP and susceptibility to this disease. Larger studies are warranted to validate our findings.

## Introduction

Coal workers' pneumoconiosis (CWP) is a lung disease caused by the inhalation and deposition of occupational coal mine dust in the lungs. The incidence and rate of CWP progression is related to the amount of respirable coal dust or silica to which coal miners were exposed during their work [1]. In China, workers exposed to coal dust or silica have increasing morbidity and mortality annually [2]. Although the exact mechanisms leading to CWP are yet to be elucidated, current evidence suggests that CWP is characterized by chronic pulmonary inflammation and fibrotic nodular lesions that usually lead to progressive fibrosis [3]. CWP can be diagnosed among workers who have worked in poor workplace environments two to three decades ago even though their work environments have been improved [4]. Therefore, identification of new genetic factors for CWP would improve diagnosis of patients at risk and help determine effective prophylactic intervention.

The *IL-4* and *IL-13* cytokines share many structural and functional similarities, as well as a common receptor component, *IL-4* receptor (*IL4R*), located on chromosome 16p11. *IL-4* plays important roles in the differentiation of T cells, eosinophilic inflammation, and isotype switching in B cells from IgM to IgE [5]. It reduces the production of proinflammatory cytokines and destructive enzymes by monocytes [6]. *IL-4* is also a key factor in the polarization of T helper cells toward Th2 differentiation [7]. *IL-13* is a central immune regulator of many allergic characteristics, including IgE synthesis, mucus hypersecretion, airway hyperreactivity, and fibrosis [8], which is found to be overexpressed in the lungs of patients and in murine models [9]. *IL-13* shares overlapping biological functions with *IL-4*, and mediates its effect through the *IL-4R* [10]. Therefore, polymorphisms in *IL-4*, *IL-4R*, and *IL-13* genes may constitute a common etiologic pathway in CWP patients. To determine whether polymorphisms in *IL-4*, *IL-4R*, and *IL-13* genes contribute to the development of CWP, we genotyped the *IL-4* C-590T (rs2243250 in the *IL-4* promoter region), *IL-4R* Ile50Val (rs1805010 in the *IL-4R* exon 5), *IL-4R* Ser478Pro (rs1805015 in the *IL-4R* exon 12), *IL-4R* Gln551Arg (rs1801275 in the *IL-4R* exon 12), *IL-13* C-1055T (rs1800925 in the *IL-13* promoter region) and *IL-13* Arg130Gln (rs20541 in the *IL-13* exon 4) polymorphisms and investigated the association between these genetic variants and CWP risk in a Chinese population.

## Materials and Methods

### Study subjects

The detailed methods of recruiting study subjects for this study have been described previously [11]. Briefly, 556 CWP patients and 541 controls were recruited in an ongoing study from five coal mines of Xuzhou Mining Business Group Co. Ltd., China, starting in January 2006. The high kilovolt chest X-ray and physical examinations were performed for reconfirming the diagnoses based on the China National Diagnostic Criteria for Pneumoconiosis (GBZ 70-2002), which is the same as the 1980 International Labour Organization (ILO) Classification of Pneumoconiosis in the judgment of opacity profusion. The pneumoconiosis patients were classified into stage I, stage II and stage III according to the size, profusion, and distribution range of opacities on chest X-ray. The chest X-rays were assessed by two independent physicians (Z Song and X Jia). The controls were selected from the same coal mines, who were matched for age (within 5 years), dust exposure period, and job type. Subjects were excluded if they had clinical evidence of autoimmunity diseases; had immunosuppressive or immunostimulatory therapy, or were subjected to radiotherapy. The epidemiological survey was done by face-to-face interviewers in order to obtain correct information including age, dust exposure period, job types, smoking status and others. The questionnaire was blinded regarding the case or control status of participants. After the interview, a 5 mL venous blood sample was collected from each subject. Informed consent was obtained from all subjects and the authorization was given by the Institutional Review Board of Nanjing Medical University.

### Genotyping

Genomic DNA was isolated from peripheral blood lymphocytes using the conventional phenol-chloroform method. Genotyping was performed using the TaqMan method with ABI 7900HT Real Time PCR system according to the manufacturer's instructions (Applied Biosystem, Foster city, CA). The sequences of primer and probe for each SNP are available on request. A total of 10 negative controls and 8 duplicates were included for each SNP as a quality control measure. About 10% of the samples were randomly selected for repeated genotyping for confirmation and the results were 100% concordant. Genotyping was performed by two persons independently in a blinded fashion.

### Statistical analyses

Differences in the distributions of demographic characteristics, selected variables, and frequencies of genotypes of *IL-4*, *IL-4R*, and *IL-13* polymorphisms between the CWP cases and controls were evaluated by using the Student's t-test (for continuous variables) or χ^2^-test (for categorical variables). Hardy-Weinberg equilibrium (HWE) was tested using a goodness-of-fit χ^2^-test. The associations between genotypes and CWP were estimated by computing odds ratios (ORs) and their 95% confidence intervals (CIs) from unconditional logistic regression analysis with the adjustment for possible confounders. For the stratified analysis, the age and dust-exposure cutoff used in this study were according to the median of age and dust-exposure year of the recruited patients and controls. The statistical power was calculated by using the PS software (http://biostat.mc.vanderbilt.edu/twiki/bin/view/Main/PowerSampleSize). EM algorithm in SAS 9.1 PROC HAPLOTYPE was used to infer haplotype frequencies based on observed genotypes. All tests were two-sided by using the SAS software (version 9.1; SAS Institute, Inc., Cary, NC), unless indicated otherwise.

### Results

The distributions of selected characteristics between CWP patients and control subjects are shown in Table 1. Briefly, there was no significant difference in the distribution of age (*P* = 0.304), exposure years (*P* = 0.452), and job types (*P* = 0.106), between the cases and controls. However, there were more ever smokers (50.9%) among the cases than among the controls (41.4%) (*P* = 0.002). Specially, light smokers (≤20 pack-years) had a 2.62-fold (95% CI = 1.93–3.57) increased risk. Furthermore, the stages from I to III of the cases were 56.7%, 34.2%, and 9.1%, respectively.

**Table 1:** Demographic and selected variables among the CWP cases and control subjects.

Variables	CWP (*n* = 556)		Controls (*n* = 541)		*P*
	N	%	N	%	
Age, year (mean ± SD)	65.3±10.5		64.8±7.2		0.304
Exposure years (mean ± SD)	26.5±9.1		26.9±8.0		0.452
Smoking status					0.002
Never	273	49.1	317	58.6	
Ever	283	50.9	224	41.4	
Former	121	21.8	25	4.6	
Current	162	29.1	199	36.8	
Pack-years smoked					<0.001
0	273	49.1	317	58.6	
0–20	183	32.9	81	15.0	
>20	100	18.0	143	26.4	
Job type					0.106
Tunnel and coal mining	525	94.4	522	96.5	
Transport	15	2.7	13	2.4	
Others	16	2.9	6	1.1	
Stage					
I	315	56.7			
II	190	34.2			
III	51	9.1			

The primary information and allele frequencies observed are listed in Table 2. All genotyped distributions of control subjects were consistent with those expected from the Hardy-Weinberg equilibrium except for Ser478Pro polymorphism in the *IL-4R* exon 12 (*P* = 0.029). The minor allele frequency (MAF) of all the six polymorphisms was consistent with that reported in the HapMap database. As shown in Table 3, only the genotype frequencies of C-590T polymorphism in the *IL-4* promoter region were significantly different between the cases and controls (*P* = 0.049 and 0.034 for genotype and allele, respectively). But this significance disappeared after the Bonferroni correction. Logistic regression analysis revealed that the *IL-4* C-590T CT genotype, but not the CC genotype, was associated with a significantly decreased risk of CWP, compared with the TT genotype (OR = 0.71, 95%CI = 0.55–0.92 for CT versus TT; and OR = 0.85, 95%CI = 0.46–1.53 for CC versus TT). Individuals with the C allele had a decreased risk of CWP, compared with those carrying the T allele (OR = 0.80, 95%CI = 0.65–0.98). Furthermore, a significant protective effect of CWP was found in the combined genotypes CT/CC, compared with the TT genotype (OR = 0.73, 95%CI = 0.57–0.94). However, no significant association with CWP was identified for the other polymorphisms examined in this study.

**Table 2:** Primary information of genotyped SNPs.

SNP	rs no.^a^	Location	Base	MAF		HWE^b^
				Case	Control	
*IL4* C-590T	rs2243250	Promoter	T>C	0.186	0.223	0.340
*IL4R* Ile50Val	rs1805010	Exon 5	C>T	0.482	0.500	0.636
*IL4R* Ser478Pro	rs1805015	Exon 12	T>C	0.081	0.087	0.029
*IL4R* Gln551Arg	rs1801275	Exon 12	A>G	0.156	0.167	0.716
*IL13* C-1055T	rs1800925	Promoter	C>T	0.159	0.165	0.690
*IL13* Arg130Gln	rs20541	Exon 4	G>A	0.272	0.300	0.054

a SNP rs no. were taken from NCBI dbSNP (http://www.ncbi.nlm.nih.gov/SNP).

b HWE *P* value in the control group.

**Table 3:** Distributions of genotypes of *IL-4*, *IL-4R* and *IL-13* their associations with risk of CWP.

Variables	CWP cases		Controls		*P* a	OR (95% CI)	OR (95% CI)^b^
	N	%	N	%			
*IL4* C-590T	*n* = 553		*n* = 541				
TT	369	66.7	323	59.7	0.049	1.00	1.00
CT	162	29.3	195	36.0		0.72 (0.56–0.93)	0.71 (0.55–0.92)
CC	22	4.0	23	4.3		0.83 (0.45–1.52)	0.85 (0.46–1.53)
CT/CC	184	33.3	218	40.3	0.016	0.74 (0.58–0.95)	0.73 (0.57–0.94)
T allele	900	81.4	841	77.7	0.034	1.00	
C allele	206	18.6	241	22.3		0.80 (0.65–0.98)	
*IL4R* Ile50Val	*n* = 556		*n* = 539				
CC	145	26.1	138	25.6	0.512	1.00	1.00
CT	286	51.4	264	49.0		1.05 (0.79–1.39)	1.06 (0.80–1.42)
TT	125	22.5	137	25.4		0.88 (0.63–1.23)	0.89 (0.64–1.25)
C allele	576	51.8	540	50.0	0.425	1.00	
T allele	536	48.2	538	50.0		0.93 (0.79–1.10)	
*IL4R* Ser478Pro	*n* = 554		*n* = 538				
TT	468	84.5	445	82.7	0.141	1.00	1.00
CT	83	15.0	93	17.3		0.85 (0.62–1.18)	0.83 (0.60–1.15)
CC	3	0.5	0	0.0		-	-
T allele	1019	92.0	983	91.4	0.606	1.00	
C allele	89	8.0	93	8.6		0.92 (0.68–1.25)	
*IL4R* Gln551Arg	*n* = 553		*n* = 534				
AA	393	71.1	372	69.7	0.756	1.00	1.00
AG	147	26.6	146	27.3		0.96 (0.74–1.26)	0.96 (0.73–1.25)
GG	13	2.3	16	3.0		0.78 (0.37–1.64)	0.78 (0.37–1.66)
A allele	933	84.4	890	83.3	0.516	1.00	
G allele	173	15.6	178	16.7		0.93 (0.74–1.17)	
*IL13* C-1055T	*n* = 552		*n* = 538				
CC	396	71.7	376	69.9	0.643	1.00	1.00
CT	137	24.8	146	27.1		0.89 (0.68–1.17)	0.90 (0.68–1.18)
TT	19	3.4	16	3.0		1.13 (0.57–2.22)	1.12 (0.57–2.22)
C allele	929	84.1	898	83.5	0.661	1.00	
T allele	175	15.9	178	16.5		0.95 (0.76–1.19)	
*IL13* Arg130Gln	*n* = 554		*n* = 539				
GG	294	53.1	255	47.3	0.131	1.00	1.00
AG	219	39.5	245	45.5		0.78 (0.61–0.99)	0.80 (0.62–1.02)
AA	41	7.4	39	7.2		0.91 (0.57–1.46)	0.94 (0.58–1.50)
G allele	807	72.8	755	70.0	0.148	1.00	
A allele	301	27.2	323	30.0		0.87 (0.72–1.05)	

a Two-sided χ^2^ test.

b Adjusted for age, exposure years, pack-years of smoking, and job type.

In the stratification analyses, we found that individuals with the *IL-4* CT/CC genotypes had a significant decreased risk of CWP than those with the TT genotype, and this decreased risk was more evident among subgroups of age <65 years (OR = 0.68, 95%CI = 0.46–0.99) and dust exposure years ≥26 years (OR = 0.69, 95%CI = 0.50–0.94) (Table 4). In addition, significant associations were observed between the genotypes and patients with stage I (OR = 0.70, 95%CI = 0.52–0.95). However, no statistical evidence was found for the gene-environment interaction (data not shown).

**Table 4:** Stratification analyses between the genotypes of *IL-4* C-590T polymorphism and CWP risk.

Variables	Cases/controls	Genotypes (cases/controls)				*P*	OR (95% CI)^a^
		TT		CT/CC			
		*n*	%	*n*	%		
Total	553/541	369/323	66.7/59.7	184/218	33.3/40.3	0.016	0.73 (0.57–0.94)
Age							
<65	214/257	147/155	68.7/60.3	67/102	31.3/39.7	0.049	0.68 (0.46–0.99)
≥65	339/284	222/168	65.5/59.2	117/116	34.5/40.8	0.130	0.78 (0.56–1.08)
Exposure years							
<26	192/182	122/107	63.5/58.5	70/75	36.5/41.2	0.397	0.83 (0.55–1.27)
≥26	361/359	247/216	68.4/60.2	114/143	31.6/39.8	0.018	0.69 (0.50–0.94)
Smoking status							
Never	272/317	180/187	66.2/59.0	187/130	33.8/41.0	0.064	0.73 (0.52–1.02)
Ever	281/224	189/136	67.3/60.7	92/88	32.7/39.3	0.116	0.74 (0.51–1.08)
Stage							
I	314/541	213/323	67.8/59.7	101/218	32.2/40.3	0.020	0.70 (0.52–0.95)
II	188/541	124/323	66.0/59.7	64/218	34.0/40.3	0.177	0.78 (0.54–1.12)
III	51/541	32/323	62.8/59.7	19/218	37.2/40.3	0.852	0.94 (0.50–1.78)

a Adjusted for age, exposure years, pack-years of smoking, and job type.

Considering potential interactions of these cytokine gene polymorphisms on risk CWP, we combined these six polymorphisms based on the numbers of variant (risk) alleles (i.e., *IL-4* rs2243250 T, *IL-4R* rs1805010 C, *IL-4R* rs1805015 T, *IL-4R* rs1801275 A, *IL-13* rs1800925 C, and *IL-13* rs20541 G alleles). As shown in Table 5, individuals with multiple risk alleles had a higher risk of CWP, compared with those with 0–8 risk alleles, with a dose-dependent manner with increasing numbers of risk variant alleles conferring increasing risk (*P*_trend_ = 0.023). Specifically, individuals carrying 11–12 risk alleles had a significantly higher risk of CWP than those with 0–8 risk alleles (OR = 1.47, 95% CI = 1.05–2.05).

**Table 5:** Frequency distributions of the combined genotypes of *IL-4*, *IL-4R* and *IL-13* between CWP cases and controls.

No. of risk alleles^a^	Cases		Controls		OR (95%CI)^b^	*P*
	No.	%	No.	%		
0–8	151	27.8	178	33.7	1.00	
9	117	21.5	112	21.2	1.25 (0.89–1.74)	0.196
10	147	27.0	136	25.8	1.28 (0.93–1.76)	0.125
11–12	129	23.7	102	19.3	1.47 (1.05–2.05)	0.025
*P*_trend_						0.023

a Risk alleles included *IL-4* rs2243250 T, *IL-4R* rs1805010 C, *IL-4R* rs1805015 T, *IL-4R* rs1801275 A, *IL-13* rs1800925 C, and *IL-13* rs20541 G alleles.

b Adjusted for age, exposure years, pack-years of smoking, and job type.

Haplotypes analysis of *IL-4R* and *IL-13* polymorphisms was performed. However, the distributions of the haplotypes of *IL-4R* and *IL-13* between the CWP cases and controls were not significantly different, and the haplotypes had no apparent relationship with risk of CWP (data not shown).

## Discussion

In this case-control study, six functional polymorphisms in the *IL-4*, *IL-4R*, and *IL-13* genes were investigated with respect to an association with risk of CWP in a Chinese population. We found that the *IL-4* C-590T polymorphism in the promoter region was significantly associated with CWP, and the association was more evident in younger workers with a long exposure history.

CWP is a chronic inflammatory lung disease where various environmental and genetic factors can influence its phenotype. Genetic factors such as polymorphisms can contribute to the extent or severity of CWP. Many genetic studies in CWP patients have involved genes encoding for cytokines and their receptors [12], [13], [14]. Cytokines play a crucial role in the widely used immunological model that explain the increasing prevalence of inflammatory diseases by an altered balance between Th1 and Th2 immune response [15]. *IL-4* is a typical Th2 cytokine of decisive significance in regulating Th1/Th2 cytokine balance [16]. It plays an important role through the *IL-4R* [17]. *IL-4R* binds not only *IL-4*, but also *IL-13*. *IL-4* is dominant mediator of Th2 cell differentiation, proliferation, and activity, whereas *IL-13* has minimal effects on T cell function [18]. A recent study reported that a promoter polymorphism C-590T of *IL-4* was associated with an increased gene expression with T allele [19]. The -590T promoter sequence showed greater binding to nuclear transcription factors from allergen stimulated Jurkat human T cells than that of -590C sequence, and alteration in electrophoretic mobility shift assay was observed [20], [21]. Several association studies have demonstrated that the T allele was correlated to inflammatory disease, such as asthma [22], [23]. In the present study, carriers of the TT genotype had an increased risk of CWP compared with the CT/CC genotypes. One possible explanation is that the T allele may result in increased anti-inflammatory cytokine production or as a pro-fibrogenic factor favoring the fibrotic nodular lesions of lung.

Polymorphisms leading to amino acid changes have been described for mouse and human *IL-4R* [24]. Three functional polymorphisms Ile50Val, Ser478Pro, and Gln551Arg in the exons of *IL-4R* are frequently linked, which were associated with low total IgE concentrations, and an increase in the phosphorylation of insulin receptor substrate molecules [25]. However, the effects of these polymorphisms on receptor signaling have been contradictory [26], [27], [28]. *IL-13* binds with high affinity to *IL-13* receptor α-1, which induces heterodimerization with *IL-4R* to form a complex identical to the type II receptor [29]. Functional characterization of *IL-13* C-1055T polymorphism showed that T allele had opposite transcriptional effects, paralleled by distinct patterns of DNA-protein interactions at the *IL-13* promoter [30], which has been shown to be associated with allergic asthma and abnormal *IL-13* production [31]. Another *IL-13* Arg130Gln polymorphism in exon 4 has been shown to be associated with high total serum IgE level [32]. However, our study found that individuals with *IL-4R* and *IL-13* polymorphisms did not have an increased risk of developing CWP. The mechanism of inconsistent results is still unknown, which needs to be validated by other studies with different ethnic populations.

Interestingly, we found that the protective effects of *IL-4* variant genotype were evident in younger workers with long exposure history. Although the exact molecular mechanisms underlying are unknown, it is possible that individuals in those subgroups more likely were less exposed to some risk factors involved in the etiology of CWP risk [33]. Hessel et al. performed a meta-analysis and showed that smoking was significantly associated with silicosis [34]. Animal studies also revealed that smoking could induce lung fibrosis [35]. In the present study, we also found a significant difference between smokers and non-smokers related to CWP risk (*P* = 0.002), but there was no difference between the smoking status and CWP patients with different *IL-4* C-590T genotypes. This negative result in our study could be due to insufficient sample size. Interestingly, we found that the *IL-4* polymorphism carriers had a significantly decreased risk of developing stage I CWP. These findings suggested that there might be different mechanisms underlying the early development of CWP and the subsequent progression of CWP [36], and the *IL-4* polymorphism might affect these two mechanisms differently.

Several limitations of this study should be addressed. First, our study was hospital-base study design, we could not rule the possible of selection bias of subjects that may have been associated with a particular genotype. Second, our sample size is moderate, and the statistical power of the study is limited, especially for subgroup and interaction analyses. However, we have 80% power at 0.05 significance level to detect an OR of 1.50 or higher and 0.63 or lower with an exposure frequency of 20% under the current sample size Third, because our results were based on the statistical significance and the ORs were very weak, the significant association between the *IL-4* C-590T polymorphism and CWP risk should be interpreted with caution. Further functional study is needed to validate our findings.

In conclusion, our present study indicated that the functional *IL4* C-590 T polymorphism is associated with decreased risk of CWP in a Chinese population. Further validation studies with diverse populations are warranted to confirm our findings.
